# Automated 16S Sequencing Using an R-Based Analysis Module for Bacterial Identification

**DOI:** 10.1128/spectrum.00408-22

**Published:** 2022-04-11

**Authors:** Kerstin Locher, Corrie R. Belanger, Eric Eckbo, Melissa Caza, Billie Velapatino, Marthe K. Charles

**Affiliations:** a Division of Medical Microbiology, Department of Pathology and Laboratory Medicine, Vancouver Coastal Health, Vancouver, British Columbia, Canada; b Department of Pathology and Laboratory Medicine, University of British Columbia, Vancouver, British Columbia, Canada; University of Cincinnati

**Keywords:** 16S Sanger sequencing, automated workflow, bacterial identification, 16S RNA, R script, automation

## Abstract

Sanger sequencing of the 16S rRNA gene is routinely used for the identification of bacterial isolates. However, this method is still performed mostly in more-specialized reference laboratories, and traditional protocols can be labor intensive. In this study, 99 clinical bacterial isolates were used to validate a fast, simplified, and largely automated protocol for 16S sequencing. The workflow combines real-time PCR of the first 500 bp of the bacterial 16S rRNA gene and amplicon sequencing on an automated, cartridge-based sequence analyzer. Sequence analysis, NCBI BLAST search, and result interpretation were performed using an automated R-based script. The automated workflow and R analysis described here produced results equal to those of manual sequence analysis. Of the 96 sequences with adequate quality, 90 were concordantly identified to the genus (*n* = 62) or species level (*n* = 28) compared with routine laboratory identification of the organism. One organism identification was discordant, and 5 resulted in an inconclusive identification. For sequences that gave a valid result, the overall accuracy of identification to at least the genus level was 98.9%. This simplified sequencing protocol provides a standardized approach to clinical 16S sequencing, analysis, and quality control that would be suited to frontline clinical microbiology laboratories with minimal experience.

**IMPORTANCE** Sanger sequencing of the 16S rRNA gene is widely used as a diagnostic tool for bacterial identification, especially in cases where routine diagnostic methods fail to provide an identification, for organisms that are difficult to culture, or from specimens where cultures remain negative. Our simplified protocol is tailored toward use in frontline laboratories with little to no experience with sequencing. It provides a highly automated workflow that can deliver fast results with little hands-on time. Implementing 16S sequencing in-house saves additional time that is otherwise required to send out isolates/specimens for identification to reference laboratories. This makes results available much faster to physicians who can in turn initiate or adjust patient treatment accordingly.

## INTRODUCTION

Timely and proficient identification of bacterial infections is crucial in clinical laboratories where delayed identification can result in increased patient morbidity and mortality. Sequence-based identification of bacterial pathogens remains a useful tool that is used routinely, especially for the identification of uncommon microorganisms or where ambiguous results are obtained by routine methods. It has proven particularly beneficial to identify bacteria directly from clinical specimens with culture-negative results (1–3).

The highly conserved bacterial 16S rRNA gene is widely used as a target, and efficient identification of bacteria to the genus or species level is achievable by sequencing the first 500 bp covering variable regions V1, V2, and V3 of this gene (2–6).

Despite the emergence of next-generation sequencing (NGS) methods, the transition to NGS in clinical microbiology laboratories has been slow and traditional Sanger 16S sequencing is still commonly used. It is, however, typically performed in specialized reference labs, and hospital microbiology laboratories have been reluctant to adopt Sanger sequencing in-house. It may be perceived as technically difficult and laborious, as traditionally, it involves conventional gel-based PCR, time-consuming maintenance of sequencing instrumentation requiring specialized staff, and manual data analysis and interpretation (7, 8).

Recently, the SeqStudio, a fully automated benchtop Sanger sequencing analyzer employing a user-friendly cartridge system requiring minimal setup time, maintenance, and technical expertise, was introduced on the market. The sequencing cartridge contains the capillaries, polymer, and reagents needed and simply clicks into the instrument, requiring minimal setup time or experience. The SeqStudio can be operated without the need for calibrations or maintenance. It is a lower throughput system, with 4 capillaries that can sequence 1 to 96 samples at a time, suited to smaller laboratories with lower sample volume and no previous experience with sequencing instrumentation. Utilizing this new automated instrument, we developed a fast and user-friendly 16S Sanger sequencing workflow for the identification of bacterial isolates that may allow frontline laboratories to implement this method. Time-saving steps in this protocol include a quick, crude DNA extraction step from bacterial isolates, the use of real-time PCR without the need for gel electrophoresis, and fast and easy sequencing run setup using the SeqStudio Sanger sequencer. This workflow is combined with fast, automated R script-based data analysis following stringent sequence quality parameters and can be completed within an 8-h shift. This method was evaluated in a centralized regional frontline microbiology laboratory on 99 bacterial isolates. Although a few groups have published fast sequencing protocols using real-time PCR or commercially available sequence analysis tools (9–11), our method is, to our knowledge, the first to combine real-time 16S PCR with a fully automated cartridge-based Sanger sequencer and automated R analysis.

## RESULTS

A total of 99 previously characterized bacterial isolates from 62 unique taxa were evaluated in this study. Of these, 88 isolates were previously identified to the species level, 9 were identified to the genus level, and 2 were identified to the family level by routine identification methods. The routine ID result was regarded as the reference result for the respective isolate. All isolates underwent extraction, amplification, and sequencing followed by manual and automated R analysis. The total time required to process, amplify, sequence, and analyze 2 isolates using this protocol with the automated R script was approximately 6.5 h, with actual hands-on time of approximately 65 min (Table 1).

**TABLE 1:** Time requirements for 16S sequencing workflow using the SeqStudio genetic analyzer

Step	Total time	Hands-on time
A. Crude DNA extraction	20 min	5 min
B. Real-time PCR	∼1–1.5 h	15 min
C. Sequencing reaction setup	∼3.5 h	∼35 min
D. Amplicon sequencing on analyzer	50 min^*a*^	∼5 min
E1. Sequence analysis using automated script	∼15 min	∼10min
E2. Sequence analysis using manual script	∼30 min	∼30 min

a Time required for 4 reactions with additional ∼40 min per each additional 4 reactions.

Generally, real-time PCR results obtained cycle threshold (*C_T_*) values of <20 (13.8 ± 3.9) and, where melt curve analysis was performed, resulted in a single melt peak. The majority of negative controls had a *C_T_* value of >30 and a melt curve with a very small peak around 87°C (87.6 ± 0.8) and/or a peak at around 75°C (75.4°C ± 1.9), which indicates primer-dimer formation and was not observed for any of the bacterial isolate PCR amplicons. Sequencing of the negative controls never resulted in any sequencing reads.

Sequencing failed in one isolate, which was excluded from the analysis. For the remaining 98 isolates, 86 resulted in good quality raw forward and reverse sequence data (i.e., both reads had a quality value [QV] of >30), and 12 had good sequencing quality for either the forward or the reverse sequence (Table 2). For the subsequent trimming and consensus sequence generation (where applicable), manual analysis using MicrobeBridge software and automated R analysis were compared (Table 2).

**TABLE 2:** Comparison of manual and automated R sequence analysis quality

Sequence type	Analysis quality of:					
	Manual sequence analysis			R analysis		
	>440 bp	400–440 bp	<400 bp	>440 bp	400–440 bp	<400 bp
Consensus sequence^*a*^ with QV > 30	83	5	0	86	6	0
Single-read sequence^*b*^ with QV > 30	3	2	5^*c*^	3	1	2^*c*^

a Consensus sequence, generated from trimmed sequences.

b After trimming.

c Sequence failed QC.

Using manual sequence analysis, after trimming, a total of 86 sequences (consensus *n* = 83, single read *n* = 3) passed QC metrics (QV >30) with a sequencing length of >440 bp and another 7 (consensus *n* = 5, single read *n* = 2) sequences passed with a slightly shorter sequencing length between 400 and 440 bp (Table 2). Using automated R analysis, 89 sequences (consensus *n* = 86, single read *n* = 3) passed QC metrics with a sequence length of >440 bp, and 7 passed QC metrics with a sequence length between 400 and 440 bp (Table 2). Overall, the automated R analysis resulted in slightly longer sequences after trimming due to minor variations in the trimming algorithm. Five sequences did not pass QC using manual analysis, and 2 samples did not pass QC using R analysis due to short sequences (<400 bp) and were excluded from BLAST analysis (Table 2).

The trimmed consensus or single-read sequences were searched against the 16S database from NCBI BLAST either manually or as part of the automated R analysis pipeline. For 6 sequences from R analysis and 8 sequences from manual analysis, the 16S database gave an inconclusive ID, and these were then searched against the standard nucleotide collection database from NCBI BLAST. This was due to a few nucleotides at the ends of the sequences that were not covered in the curated 16S sequences but aligned with the longer reference sequences in the nucleotide database.

Using manual analysis, of the 93 sequences with adequate QC metrics, 85 were concordantly identified to the genus (*n* = 57) or species level (*n* = 28) compared to the routine laboratory identification (Table 3, see Table S1 for detailed results). Seven sequences resulted in inconclusive identification due to failed QC (query cover <98%, *n* = 5; percent identity <97%, *n* = 2), and one ID was discordant from the reference result. Overall, 85 out of 86 valid ID results were concordant with the reference result for an agreement of 98.8%.

**TABLE 3:** Overview of 16S sequencing results comparing manual analysis to automated analysis using R

Characteristic	Value for:	
	Manual sequence analysis	R analysis
Initial sequence QC met	93	96
Excluded from analysis (poor sequence QC)	5	2
Identification concordant to genus or group level	57	62
Identification concordant to species level	28	28
Identification discordant with reference result	1	1
Identification inconclusive	7	5

The performance of the automated R analysis script was equal to manual sequence analysis. Of the 96 samples with adequate QC, 5 sequences resulted in inconclusive identification due to one or more QC not met (query cover < 98%, *n* = 2; % identity < 97%, *n* = 1, short sequence, *n* = 2). Of the remaining 91 samples, one ID was discordant from the reference result and 90 were concordantly identified to either genus/group level (*n* = 62) or species level (*n* = 28) (Table 3) for an overall agreement of 98.9% with the reference result.

One discordant result, for which the reference result was reported as *Propionibacteriaceae* family, was identified as Staphylococcus lentus by 16S sequencing using manual and R analysis, with all QC criteria met. The original culture had two morphologies described, and the discordant result may have originated from two different organisms present in a mixed culture.

Overall, 3 isolates were identified to species level using manual analysis and to genus level or inconclusive using R analysis. Similarly, there were 3 isolates identified to species level by R analysis but not by manual analysis (Table 4). An additional 6 isolates that were resolved by R analysis to genus level could not be resolved or did not meet QC using the manual analysis settings (query cover < 98%, *n* = 4; percent identity < 97%, *n* = 1; sequence alignment < 400 bp, *n* = 1), and 2 isolates were resolved manually to genus but not resolved by R analysis (percent identity < 97%, *n* = 1; sequence alignment < 400 bp, *n* = 1). No discordant identifications were observed between the two methods.

**TABLE 4:** Overview of results with different final ID resolution between manual and R analysis

Reference method ID	Manual BLAST analysis					Automated R analysis				
	16S result	Aligned (bp)	Query cover	% id	% distance^*a*^	16S result	Aligned (bp)	Query cover	% id	% distance^*a*^
*Selenomonas* sp.	Inconclusive	440	100	95.7	2.0	Selenomonas infelix	509	99	99.5	1.5
Fusobacterium necrophorum	Inconclusive	457	97	98.0	0.9	*Fusobacterium* sp.	454	99	98.7	2.0
Acinetobacter seifertii	Inconclusive	478	97	99.8	0.9	Acinetobacter sp.	487	99	98.4	0.2
Burkholderia cenocepacia	Inconclusive	478	97	99.6	0	*Burkholderia* sp.	487	99	98.8	0
Butyricimonas virosa	Inconclusive	478	99	96.9	1.7	*Butyricimonas* sp.	496	100	97.8	3.0
Comamonas kerstersii	Inconclusive	462	96	98.9	2.1	*Comamonas* sp.	488	99	99.0	0.7
Clostridium perfringens	C. perfringens	456	98	99.6	5.0	*Clostridium* sp.	479	99	98.5	5.0
Corynebacterium diphtheriae	C. diphtheriae	450	100	99.8	3.4	Inconclusive	427	98	98.9	0.5
Neisseria gonorrhoeae	N. gonorrhoeae	482	98	99.6	2.2	*Neiserria* sp.	494	99	98.5	2.3
Pseudomonas protegens	Pseudomonas sp.	429	98	99.8	1.6	P. protegens	478	100	99.4	2.0
Streptococcus pneumoniae	S. mitis group	412	100	99.3	0.2	Inconclusive	395	100	100	0.3
*Parvimonas* sp.	*Parvimonas* sp.	466	98	98.7	NA	Inconclusive	476	98	96.2	NA
*Fusobacterium species*	Excluded	399	100	99.8	1.4	F. nucleatum	400	100	99.5	1.5
Bacteroides thetaiotaomicron	Excluded	381	99	99.0	0.3	*Bacteroides* sp.	400	100	98.9	2.9

a To next species.

## DISCUSSION

Despite 16S Sanger sequencing being widely used for the identification of bacteria from isolates or clinical specimens, this method is often performed in reference laboratories. Reluctance of frontline microbiology laboratories to adopt Sanger sequencing may stem from the fact that historically, this method has been regarded as laborious and requiring specialized personnel (8, 12). Here, we present an improved and more automated workflow for the fast and user-friendly 16S sequencing of bacterial isolates that combines the advantages of crude nucleic acid extraction and real-time PCR with a user-friendly cartridge-based Sanger sequencing platform and automated R-based sequence analysis. This protocol is tailored toward the implementation in frontline microbiology laboratories.

Traditionally, presequencing analysis of PCR amplicons uses gel electrophoresis to determine if appropriate bands are present and of sufficient quality (9, 13, 14). Previously, a few studies have demonstrated that real-time PCR can be used to efficiently amplify the 16S target without the need for quantification or electrophoresis prior to sequencing (3, 15, 16). We used real-time PCR along with melt curve analysis to examine the quality of the amplicons. While developing this method, it was our experience that, generally, amplicons are of high quality, and the sequencing workflow can be completed faster by excluding a melt curve analysis without affecting results. The use of a new cartridge-based 4-capillary Sanger sequencer that can be operated by an inexperienced user further simplifies this protocol. The instrument does not require elaborate run setup or maintenance compared to previous Sanger sequencing systems and is able to generate 4 sequences every 40 min. The sequence files are then immediately available for data analysis while the next set of samples is being processed. While this instrument has a lower throughput, its user-friendliness makes it particularly useful for smaller laboratories.

While there are a few commercially available automated 16S sequence analysis-based microbial identification services available (2, 17, 18), these can add significant cost per sample.

We aimed to eliminate labor-intensive sequence data analysis and interpretation by using a custom R script that assigns quality values, trims reads, generates consensus sequences, and subsequently aligns the consensus sequences against the NCBI 16S database. Rigorous quality control criteria for sequence data analysis and interpretation were followed to guide the sequence-based identification of bacterial isolates. Sequence quality cutoffs and distance to next species were based on CLSI guidelines (19, 20) following a transparent and straightforward QC algorithm.

When evaluated on clinical isolates, results from the manual analysis method and the automated R analysis method were in high agreement with the reference results. Of note, the majority of isolates were identified to the genus level. This is a combination of the inherently limited discrimination power of sequencing the first 500 bp of the 16S gene within many genera (2, 19) and strict QC metrics that were followed in this algorithm for sequence data analysis. Many organisms that were resolved to the genus level were correctly identified to the species level; however, not all QC criteria were met for reporting a species. As stated in the CLSI guidelines, results from 16S sequencing should always be reviewed and considered in conjunction with results from other phenotypical testing or matrix-assisted laser desorption ionization (MALDI) where available. In such cases, a result may be reported as the genus with a comment “most closely related to *species*” (19). In most cases, this will provide sufficient information to make an appropriate clinical decision.

Overall, the automated R analysis performed slightly better, with 3% more reads passing sequencing QC and 6% more valid identifications to the genus or species level than manual analysis. This was likely due to the trimming algorithm used in Sangeranalyse.R, which provided slightly longer sequences and often less-ambiguous base calls than the manual analysis method. Notably, the automated consensus sequence generation and BLAST analysis considerably decreased hands-on time for each sample. Both methods gave one discordant result for the same isolate, which was likely due to a mixed culture.

Some limitations that might be considered when using this automated analysis of 16S sequencing data, include the inability to visualize and edit basecalls using the Sangeranalyse.R trimming algorithm. Furthermore, the NCBI 16S and nucleotide databases for blast alignments need to be downloaded onto a local computer, which requires computational space and regular updating.

We demonstrated in this study that this protocol, combining a fast and easy-to-use sequencing workflow using a simplified sequence analyzer with automated sequence analysis, allows 16S sequencing with a quick turnaround time and accurate results and might reduce reluctance for frontline laboratories to implement in-house 16S sequencing.

## MATERIALS AND METHODS

### Isolates and conventional identification methods.

A panel of 99 previously characterized bacterial isolates representing a diverse set of taxa were included in this study. Routine identification of isolates was performed by matrix-assisted laser desorption ionization–time of flight mass spectrometry (MALDI-TOF; MALDI Biotyper; Bruker, MA, USA) and/or conventional biochemical methods or by sequencing of the first 500 bp of the bacterial 16S rRNA gene at the British Columbia Center for Disease Control (BCCDC) (21).

### Extraction and amplification.

Bacterial isolates underwent fast, crude DNA extraction with bead beating. Cells were suspended in 500 μL H_2_O and approximately 50 μL of 0.1-mm glass beads (BioSpec Products, Inc., Bartlesville, OK, USA), incubated for 15 min at 100°C, and then vortexed at high speed for 5 min using a Disruptor Genie (Scientific Industries, Bohemia, NY). The primers used in this study were 16S-dual priming oligonucleotide (DPO) primers with the forward sequence AGAGTTTGATCMTGGCTCA-I-I-I-I-I-AACGCT and the reverse sequence CGCGGCTGCTGGCA-I-I-I-A-I-TTRGC (15), targeting the first 500 bp of the bacterial 16S rRNA gene. PCRs were prepared in 20 μL volumes with Luna Universal qPCR master mix (New England Biolabs Inc., MA USA), 0.4 μM concentrations of the forward and reverse primers, and 3 μL of bacterial lysate. Amplification was performed on an ABI 7500 Fast real-time thermocycler (Thermo Fisher Scientific Inc., MA) using a thermocycling profile as described previously (9, 15). Where melt curve analysis was performed, the Applied Biosystems high-resolution melting protocol was followed.

### 16S sequencing.

PCR products were cleaned using ExoSAP-IT Express PCR product cleanup (Thermo Fisher Scientific) following manufacturer’s instructions and diluted 1:10 in sterile molecular-grade water. Amplicon sequencing was performed bidirectionally, with separate forward and reverse reactions, using the BigDye Terminator v3.1 cycle sequencing kit (Thermo Fisher Scientific), and reactions were purified with the BigDye XTerminator purification kit (Thermo Fisher Scientific). Manufacturer instructions were followed for each kit. Sequencing reactions were analyzed on a SeqStudio genetic analyzer using a SeqStudio Cartridge v1 (Thermo Fisher Scientific) with a 10-s injection time, dye set of Z BigDye Terminator v3.1, and a mediumseq module setting. Negative-control samples that underwent the extraction procedure, real-time PCR, and sequencing as well as a manufacturer-provided sequencing positive control were included in each run.

### Sequence analysis and identification.

Analysis of 16S sequences was performed with stringent quality control (QC) criteria following the 1st and 2nd editions of the Clinical and Laboratory Standards Institute (CLSI) MM18 sequencing guidelines (19, 20) and methods previously published by others (22, 23).

Manual sequence trimming and consensus sequence generation were performed using the MicrobeBridge analysis software (ThermoFisher Scientific) (24) with the following settings: trimming cutoffs were set to <15% of bp with a quality value (QV) of >30 and maximum undetermined bases at 10%, secondary peak cutoff was set to 0.33, minimum clear length was set to 50, and maximum mixed bases was set to 20. Consensus sequences were searched against the National Center for Biotechnology Information (NCBI) nucleotide Basic Local Alignment Search Tool (BLAST) using the nucleotide rRNA/RefSeq targeted loci project database (16S database) (25). In addition, automated analysis was performed with a custom R analysis (26) script and standalone BLAST+ (25). Using the script, all forward and reverse ab1 files from a sequencing run were simultaneously trimmed for quality and aligned into consensus sequences utilizing SangerAnalyse.R (27) with an average trimming cutoff of QV = 40 over a sliding window of 10 bp, a signal-to-noise ratio of 0.33, and a minimum clear length of 20. Contig alignments of forward and reverse reads, sequence qualities, and fasta consensus sequences were exported automatically for review. Consensus sequences were then automatically searched against a local 16S database downloaded from the NCBI BLAST website using a custom script that searched local BLAST+. For both analysis methods, if a sequence resulted in inconclusive identity when searched against the 16S database, a broader search was done against the nucleotide collection. An overview of QC metrics used to identify specimens to the species or genus level is shown in Table 5. The R script used for this workflow is available at https://github.com/CorrieRB/16S_ITS_Analysis.git.

**TABLE 5:** 16S quality control parameters for identification of bacterial pathogens

Parameter	Final identification				
	To species	To genus	To genus	Inconclusive	Inconclusive
Distance to next species^*a*^	≥0.8%	<0.8%	NA	NA	NA
% identity to reference sequence^*a*^	≥99%	≥99%	97–99%	<97%	NA
Query cover^*a*^	≥98%	≥98%	≥98%	≥98%	<98%
Aligned query length^*a*^	>440 bp^*b*^	>440 bp^*b*^	>440 bp^*b*^	>440 bp^*b*^	>440 bp^*b*^

a In NCBI BLAST database.

b If aligned query length was between 400 and 440 base pairs, sequences were identified to genus if query cover was ≥98 and % identity was ≥99%.

### Data interpretation.

The final identification obtained by 16S sequencing was compared to the reference identification that was reported for the respective isolate. An identification was considered concordant with the reference result if an isolate was correctly identified to at least the genus level. An inconclusive identification was not regarded as discordant but was omitted from the calculation of agreement (Table 3).

## References

[1] Stavnsbjerg C, Frimodt-Møller N, Moser C, Bjarnsholt T. 2017. Comparison of two commercial broad-range PCR and sequencing assays for identification of bacteria in culture-negative clinical samples. BMC Infect Dis 17:233. doi:10.1186/s12879-017-2333-9. 28347280 PMC5368927

[2] Church DL, Cerutti L, Gürtler A, Griener T, Zelazny A, Emler S. 2020. Performance and application of 16S rRNA gene cycle sequencing for routine identification of bacteria in the clinical microbiology laboratory. Clin Microbiol Rev 33. doi:10.1128/CMR.00053-19. PMC7484979 32907806

[3] Wagner K, Springer B, Pires VP, Keller PM. 2019. High-throughput screening of bacterial pathogens in clinical specimens using 16S rDNA qPCR and fragment analysis. Diagn Microbiol Infect Dis 93:287–292. doi:10.1016/j.diagmicrobio.2018.11.006. 30545581

[4] Fadrosh DW, Ma B, Gajer P, Sengamalay N, Ott S, Brotman RM, Ravel J. 2014. An improved dual-indexing approach for multiplexed 16S rRNA gene sequencing on the Illumina MiSeq platform. Microbiome 2:6. doi:10.1186/2049-2618-2-6. 24558975 PMC3940169

[5] Watts GS, Youens-Clark K, Slepian MJ, Wolk DM, Oshiro MM, Metzger GS, Dhingra D, Cranmer LD, Hurwitz BL. 2017. 16S rRNA gene sequencing on a benchtop sequencer: accuracy for identification of clinically important bacteria. J Appl Microbiol 123:1584–1596. doi:10.1111/jam.13590. 28940494 PMC5765505

[6] Peker N, Garcia-Croes S, Dijkhuizen B, Wiersma HH, van Zanten E, Wisselink G, Friedrich AW, Kooistra-Smid M, Sinha B, Rossen JWA, Couto N. 2019. A comparison of three different bioinformatics analyses of the 16S-23S rRNA encoding region for bacterial identification. Front Microbiol 10:620. doi:10.3389/fmicb.2019.00620. 31040829 PMC6476902

[7] Clarridge JE, 3rd. 2004. Impact of 16S rRNA gene sequence analysis for identification of bacteria on clinical microbiology and infectious diseases. Clin Microbiol Rev 17:840–862. doi:10.1128/CMR.17.4.840-862.2004. 15489351 PMC523561

[8] Woo PCY, Lau SKP, Teng JLL, Tse H, Yuen K-Y. 2008. Then and now: use of 16S rDNA gene sequencing for bacterial identification and discovery of novel bacteria in clinical microbiology laboratories. Clin Microbiol Infect 14:908–934. doi:10.1111/j.1469-0691.2008.02070.x. 18828852

[9] Miller RJH, Chow B, Pillai D, Church D. 2016. Development and evaluation of a novel fast broad-range 16S ribosomal DNA PCR and sequencing assay for diagnosis of bacterial infective endocarditis: multi-year experience in a large Canadian healthcare zone and a literature review. BMC Infect Dis 16:146. doi:10.1186/s12879-016-1476-4. 27066823 PMC4828839

[10] Krohn S, Böhm S, Engelmann C, Hartmann J, Brodzinski A, Chatzinotas A, Zeller K, Prywerek D, Fetzer I, Berg T. 2014. Application of qualitative and quantitative real-time PCR, direct sequencing, and terminal restriction fragment length polymorphism analysis for detection and identification of polymicrobial 16S rRNA genes in ascites. J Clin Microbiol 52:1754–1757. doi:10.1128/JCM.00552-14. 24622095 PMC3993693

[11] Vliegen I, Jacobs JA, Beuken E, Bruggeman CA, Vink C. 2006. Rapid identification of bacteria by real-time amplification and sequencing of the 16S rRNA gene. J Microbiol Methods 66:156–164. doi:10.1016/j.mimet.2005.11.005. 16371239

[12] Renvoise A, Brossier F, Sougakoff W, Jarlier V, Aubry A. 2013. Broad-range PCR: past, present, or future of bacteriology? Med Mal Infect 43:322–330. doi:10.1016/j.medmal.2013.06.003. 23876208

[13] Cherkaoui A, Emonet S, Ceroni D, Candolfi B, Hibbs J, Francois P, Schrenzel J. 2009. Development and validation of a modified broad-range 16S rDNA PCR for diagnostic purposes in clinical microbiology. J Microbiol Methods 79:227–231. doi:10.1016/j.mimet.2009.09.014. 19782706

[14] Cherkaoui A, Hibbs J, Emonet S, Tangomo M, Girard M, Francois P, Schrenzel J. 2010. Comparison of two matrix-assisted laser desorption ionization-time of flight mass spectrometry methods with conventional phenotypic identification for routine identification of bacteria to the species level. J Clin Microbiol 48:1169–1175. doi:10.1128/JCM.01881-09. 20164271 PMC2849558

[15] Kommedal Ø, Simmon K, Karaca D, Langeland N, Wiker HG. 2012. Dual priming oligonucleotides for broad-range amplification of the bacterial 16S rRNA gene directly from human clinical specimens. J Clin Microbiol 50:1289–1294. doi:10.1128/JCM.06269-11. 22278843 PMC3318507

[16] Christensen JJ, Andersen K, Justesen T, Kemp M. 2005. Ribosomal DNA sequencing: experiences from use in the Danish National Reference Laboratory for Identification of Bacteria. APMIS 113:621–628. doi:10.1111/j.1600-0463.2005.apm_224.x. 16218938

[17] Kommedal Ø, Lekang K, Langeland N, Wiker HG. 2011. Characterization of polybacterial clinical samples using a set of group-specific broad-range primers targeting the 16S rRNA gene followed by DNA sequencing and RipSeq analysis. J Med Microbiol 60:927–936. doi:10.1099/jmm.0.028373-0. 21436365 PMC3168215

[18] Simmon KE, Croft AC, Petti CA. 2006. Application of SmartGene IDNS software to partial 16S rRNA gene sequences for a diverse group of bacteria in a clinical laboratory. J Clin Microbiol 44:4400–4406. doi:10.1128/JCM.01364-06. 17050811 PMC1698390

[19] Clinical and Laboratory Standards Institute. 2018. Interpretive criteria for identification of bacteria and fungi by targeted DNA sequencing, 2nd ed. CLSI Guideline MM18. Clinical and Laboratory Standards Institute, Wayne, PA.

[20] Clinical and Laboratory Standards Institute. 2008. Interpretive criteria for identification of bacteria and fungi by targeted DNA sequencing; approved guideline. CLSI Guideline MM18-A. Clinical and Laboratory Standards Institute, Wayne, PA.

[21] Payne M, Azana R, Hoang LM. 2016. Review of 16S and ITS direct sequencing results for clinical specimens submitted to a reference laboratory. Can J Infect Dis Med Microbiol 2016:4210129. 27366168 10.1155/2016/4210129 PMC4904561

[22] Drancourt M, Bollet C, Carlioz A, Martelin R, Gayral JP, Raoult D. 2000. 16S ribosomal DNA sequence analysis of a large collection of environmental and clinical unidentifiable bacterial isolates. J Clin Microbiol 38:3623–3630. doi:10.1128/JCM.38.10.3623-3630.2000. 11015374 PMC87447

[23] Kommedal O, Karlsen B, Saebo O. 2008. Analysis of mixed sequencing chromatograms and its application in direct 16S rRNA gene sequencing of polymicrobial samples. J Clin Microbiol 46:3766–3771. doi:10.1128/JCM.00213-08. 18768654 PMC2576573

[24] Thermo Fisher Scientific. 2015. MicrobeBridge Software v1.0. Thermo Fisher Scientific, Waltham, MA.

[25] McGinnis S, Madden TL. 2004. BLAST: at the core of a powerful and diverse set of sequence analysis tools. Nucleic Acids Res 32:W20–W25. doi:10.1093/nar/gkh435. 15215342 PMC441573

[26] Team RC. 2021. R: a language and environment for statistical computing. R Foundation for Statistical Computing, Vienna, Austria.

[27] Chao KH, Barton K, Palmer S, Lanfear R. 2021. sangeranalyseR: simple and interactive processing of Sanger sequencing data in R. Genome Biol Evol 13. doi:10.1093/gbe/evab028. PMC7939931 33591316

